# Robust Gaussian and Nonlinear Hybrid Invariant Clustered Features Aided Approach for Speeded Brain Tumor Diagnosis

**DOI:** 10.3390/life12071084

**Published:** 2022-07-20

**Authors:** Yassir Edrees Almalki, Muhammad Umair Ali, Waqas Ahmed, Karam Dad Kallu, Amad Zafar, Sharifa Khalid Alduraibi, Muhammad Irfan, Mohammad Abd Alkhalik Basha, Hassan A. Alshamrani, Alaa Khalid Alduraibi

**Affiliations:** 1 Division of Radiology, Department of Internal Medicine, Medical College, Najran University, Najran 61441, Saudi Arabia; yealmalki@nu.edu.sa; 2 Department of Unmanned Vehicle Engineering, Sejong University, Seoul 05006, Korea; umair@sejong.ac.kr; 3 Secret Minds, Entrepreneurial Organization, Islamabad 44000, Pakistan; engnr.waqasahmed@gmail.com; 4 Department of Robotics and Intelligent Machine Engineering (RIME), School of Mechanical and Manufacturing Engineering (SMME), National University of Sciences and Technology (NUST), H-12, Islamabad 44000, Pakistan; karamdad.kallu@smme.nust.edu.pk; 5 Department of Electrical Engineering, The Ibadat International University, Islamabad 54590, Pakistan; 6 Department of Radiology, College of Medicine, Qassim University, Buraidah 52571, Saudi Arabia; salduraibi@qu.edu.sa (S.K.A.); al.alderaibi@qu.edu.sa (A.K.A.); 7 Electrical Engineering Department, College of Engineering, Najran University, Najran 61441, Saudi Arabia; miditta@nu.edu.sa; 8 Radiology Department, Faculty of Human Medicine, Zagazig University, Zagazig 44631, Egypt; maatya@zu.edu.eg; 9 Radiological Sciences Department, College of Applied Medical Sciences, Najran University, Najran 61441, Saudi Arabia; hamalshamrani@nu.edu.sa

**Keywords:** magnetic resonance imaging (MRI), brain tumor, machine learning

## Abstract

Brain tumors reduce life expectancy due to the lack of a cure. Moreover, their diagnosis involves complex and costly procedures such as magnetic resonance imaging (MRI) and lengthy, careful examination to determine their severity. However, the timely diagnosis of brain tumors in their early stages may save a patient’s life. Therefore, this work utilizes MRI with a machine learning approach to diagnose brain tumor severity (glioma, meningioma, no tumor, and pituitary) in a timely manner. MRI Gaussian and nonlinear scale features are extracted due to their robustness over rotation, scaling, and noise issues, which are common in image processing features such as texture, local binary patterns, histograms of oriented gradient, etc. For the features, each MRI is broken down into multiple small 8 × 8-pixel MR images to capture small details. To counter memory issues, the strongest features based on variance are selected and segmented into 400 Gaussian and 400 nonlinear scale features, and these features are hybridized against each MRI. Finally, classical machine learning classifiers are utilized to check the performance of the proposed hybrid feature vector. An available online brain MRI image dataset is utilized to validate the proposed approach. The results show that the support vector machine-trained model has the highest classification accuracy of 95.33%, with a low computational time. The results are also compared with the recent literature, which shows that the proposed model can be helpful for clinicians/doctors for the early diagnosis of brain tumors.

## 1. Introduction

The brain is the most complex organ in the human body. It has over 100 billion nerve cells with trillions of synapses [1]. In other words, the human brain is the primary command and control center of the neurological system. Therefore, an injury in the brain has a catastrophic influence on human health. For example, in a brain tumor, the development of abnormal brain cells may damage the brain and may even threaten a patient’s life. Because brain tumors have long-term and life-altering physical and psychological implications, they can significantly influence a patient’s living quality and affect their entire life [2]. According to a World Health Organization (WHO) report [3], cancer is the second greatest cause of mortality globally. It is responsible for around 10 million fatalities. Therefore, early cancer identification improves the patient’s survival chances. According to a National Brain Tumor Foundation (NBTF) report [4], around 29,000 persons in the USA have primary malignant tumors, and 13,000 people die due to this type of brain tumor. 

The location, progression stage, type, and rate of growth of brain tumors determine whether they are benign or malignant [5,6]. The affected cells rarely attack nearly healthy cells in benign brain tumors. They also progress slowly and have clear limits, such as in meningioma and pituitary tumors. In contrast, neighboring healthy cells are influenced by affected cells in malignant brain tumors. These tumors also have a fast advancement rate with broad limitations, such as gliomas. Furthermore, brain tumors may be divided into two types based on their origin: primary and secondary brain tumors [7]. The brain tumors that start in the brain tissues are known as primary tumors. In contrast, secondary brain tumors develop in many areas of the central nervous system (CNS) and move to the brain via the blood vessels. Therefore, early cancer type detection (meningioma, pituitary, and glioma) is crucial for cancer treatment to save the patient’s life.

For brain tumor detection, several diagnostic methods, both invasive and non-invasive, are utilized [8]. A biopsy is an invasive approach: a sample is retrieved by an incision and is inspected under a microscope to assess malignancy. Unlike other tumors in other areas of the body, the biopsy is usually delayed until the final brain surgery. Due to this, computer-aided diagnostics (CAD) (non-invasive) such as computed tomography (CT), positron emission tomography (PET), and magnetic resonance imaging (MRI) are thought to be faster and safer than a biopsy for diagnosing brain tumors. Brain MRI is considered to be the most recommended method owing to its ability to provide extensive information regarding the position, extension, nature, and size of the brain tumor [9]. Meanwhile, manual MRI scan interpretation takes a long time and has a significant risk of mistakes. Therefore, an automatic computer-aided diagnostic approach is required for injury detection in the brain.

The evolution of machine learning methods has increased CAD systems’ efficiency in assisting doctors in identifying brain tumors [7,10,11]. Numerous learning methods have been presented in the literature to diagnose brain tumors; they can be further categorized as deep learning and classical learning methods based on the literature [12]. In deep learning approaches, convolution neural networks (CNNs) are generally utilized to identify brain tumors using MRI [13]. Various researchers have used pre-trained and developed learning models to classify MRI images. In one work [14], the authors developed a CNN model to classify brain MRI images into two classes (tumor and no tumor). The main shortcoming of their model was the detection of the subclasses of the tumor. Abiwinanda et al. [15] designed a CNN model to detect brain tumor subclasses (glioma, meningioma, and pituitary). However, their model had a low accuracy of only 84.19%. Recently, a new CNN model was developed to classify brain MRI images into three subclasses [8]. The authors also performed data augmentation to enhance the classification accuracy of brain MRI images. A classification accuracy of 96.56% was achieved using a 10-fold cross-validation approach. Irmak [16] developed a 25-layer CNN model to classify brain images into five classes, with an accuracy of 92.66%. Pre-trained networks such as GoogLeNet and ResNet-50 are also used to classify brain images [17,18,19]. However, the deep networks require long training times, have a complex architecture, high memory requirements, a strong processing unit (GPU), etc.

In contrast to deep learning models, classical models require the most basic features of brain MRI images to diagnose a brain tumor. Therefore, they require less time to train the models; methods include support vector machine (SVM), tree, Naïve Bayes, etc. Kumari et al. [20] computed the gray-level co-occurrence matrix of brain MRI images to classify them into two classes. The model’s accuracy was high; however, the authors only detected the tumors on the brain MRI images. The accuracy of these global-level features is not high due to the high similarity in the brain MRI images. Therefore, local-level features such as the bag of words [21], Fisher vector [22], and scale-invariant feature transformation [23] are also used to classify brain MRI images. In one study [24], the authors hybridized the gray-level co-occurrence matrix, histogram intensity, and bag of words to classify brain MRI images. They achieved a classification accuracy of 91.28% for the three-class classification MRI dataset. In a recent study [25], the authors calculated the deep features of brain MRI image datasets using pre-trained CNN models. The results showed that the hybrid features of the pre-trained model had the best accuracy of 93.72% when using an SVM classifier. However, the size of their dataset was large, and it required a long training time. Moreover, in machine learning images/MRI feature extraction approaches, features such as texture (extracted through gray-level co-occurrence matrix), local binary pattern, histogram of oriented gradient, etc., are quite sensitive to noise, scaling, rotation, visibility, etc., which affect the performance, memory requirement, execution time, etc.

Considering the shortcomings of deep and machine learning approaches, the following are the main contributions of this work:

1. This study presents a fast automatic approach for brain tumor detection and differentiation using brain MRI images to increase the accuracy, grading, robustness to noise, rotation, and scaling with the least memory and processing system requirements.
2. The Gaussian scale-space features are extracted through speeded up robust features (SURF) and nonlinear scale-space features are extracted through KAZE of brain MRI images.
3. Each MRI is divided into sub-MRIs of 8 × 8-pixel images to capture the small details/tumor information.
4. Afterwards, to reduce the memory requirements, the strongest features are selected based on variance and subjected to segmentation into 400 Gaussian features and 400 nonlinear features against each brain MRI scan (a total of 800 features).
5. Various classical machine learning models are trained to check their performance.
6. Finally, two available online datasets are used to validate the proposed approach.
7. The findings of the work are also compared with the approaches present in the literature.

The paper’s organization is as follows: Section 2 presents the feature extraction and the workings of the proposed approach. Then, the dataset and results are presented in the third section. Finally, the results are discussed and concluded in Section 4 and Section 5.

## 2. Materials and Methods

### 2.1. Feature Extraction 

In computer image processing, feature detection and description are hot topics. In image classification applications, computing features that are repeatable and distinct in the face of various image transformations are of high importance. The classification of brain tumors also mainly relies on retrieving the relevant and relatable features from brain MRI images. Therefore, many global [20] and local features [22,23] are used to classify brain MRI images. The global-level features have accuracy problems in a multiclass environment, as discussed in Section 1. Various local features such as scale-invariant feature transform (SIFT) [26], speeded up robust features (SURF) [27], and KAZE [28] compute distinctive features at various interest point locations. These distinctive features primarily relate to the local maxima/minima/mean in regard to the computed feature. A descriptor vector represents the intensity patterns surrounding these interest points. Lowe [26] introduced the SIFT feature descriptor. It gained much attention owing to its translation invariance, robustness to image noise, invariance to scale, and rotation invariance properties. However, the computational cost of SIFT feature extraction is very high, so it is not recommended for real-time applications [29]. 

#### 2.1.1. Speeded up Robust Feature (SURF)

To overcome the issues related to SIFT, Bay et al. [27] introduced the SURF method to tackle the robustness issues of the SIFT approach. The SURF approach is based on Gaussian scale-space image analysis, similar to the SIFT method. Unlike the SIFT detector, the SURF approach depends on the Hessian Matrix determinant. It employs integrated images to enhance the speed of feature detection. SURF’s 64-bin descriptor characterizes each detected feature using a dispersion of Haar wavelet responses within a specific area. Unlike SIFT, the SURF features show limited affine invariance. However, to deal with more considerable viewpoint shifts, the descriptor can be expanded to 128-bin values. The Hessian Matrix is generated at the point “m=(m,n)” at scale “σ”.

(1) $$H(m,\sigma )=\left[\begin{array}{cc} L_{mm}(m,\sigma ) & L_{mn}(m,\sigma )\\ L_{mn}(m,\sigma ) & L_{nn}(m,\sigma ) \end{array}\right]$$

where $L_{mm}(m,\sigma )$ is the Gaussian second-order derivate convolution $\frac{\partial^{2}}{\partial x^{2}}g(\sigma )$ with the image I at a point m, similar to $L_{mn}(m,\sigma )$ and $L_{nn}(m,\sigma )$.

#### 2.1.2. KAZE

KAZE is a revolutionary 2D feature identification and description approach that works entirely in nonlinear scale-space using nonlinear diffusion and the additive operator splitting method [28]. Thus, blurring in images becomes locally adaptable to feature points, resulting in noise reduction without affecting the image region boundaries. The KAZE is derived by the Hessian Matrix determinant with a normalized scale and is calculated at different scale levels. A moving window identifies the maxima/minima/mean of detector response as feature points (mean is used in this work). In the feature description, the rotation invariance property is introduced by determining the prevalent orientation in a rounded region surrounding each detected feature. It has the properties of scale and rotation invariance, little invariance to affine, and has greater distinctness at different scales, with a slight increase in computational cost. The nonlinear diffusion equation is presented below.

(2) $$\frac{\partial L}{\partial t}=div\left(c\left(m,n,t\right).\nabla L\right)$$

where c, div, ∇, and L are the conductivity function, divergence, gradient operator, and luminance of the image, respectively. 

### 2.2. Support Vector Machine (SVM)

Cortes and Vapnik [30] proposed the SVM model in 1995, and it is a very popular and powerful classifier used in various fields [31,32,33]. The SVM algorithm uses kernel functions $K(x,x_{a})$ to transfer the nonlinear low-dimensional input data space into a high-dimensional linear data space. The hyperplane function used to separate the transferred data (high-dimensional linear data) is presented in Equation (3).

(3) $$y(x)=\sum_{a=1}^{n}\beta_{a}K(x,x_{a})+b_{1}$$

Meanwhile, various kernel functions, such as linear kernel, sigmoid kernel, and RBF kernel, can be used to classify the data. Further details about SVM can be found in [30,32].

### 2.3. Proposed Framework

**Figure 1:**
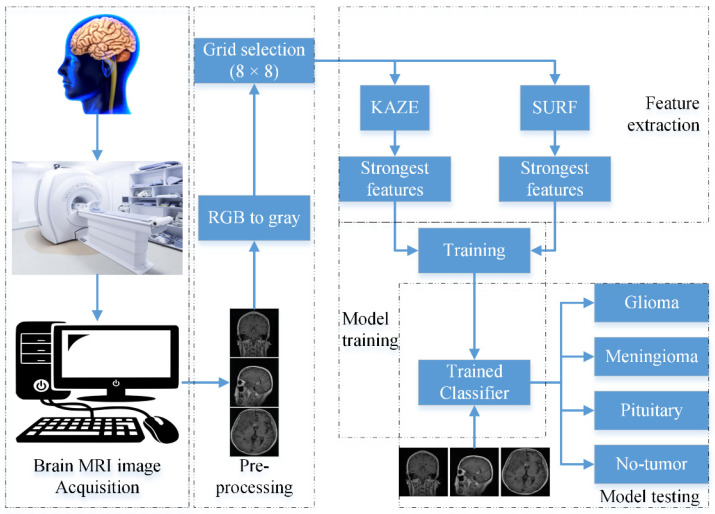
Framework of the proposed hybrid brain MRI image classification model.

This section discusses the overall framework of the proposed approach in detail. The proposed approach consists of 4 main components, namely brain MRI image acquisition, pre-processing, feature extraction, and model training, as shown in Figure 1.

The brain images were acquired using the brain MRI machine in the first step. Next, the acquired brain MRI images were pre-processed from the RGB images into grayscale images. Then, an 8 × 8-pixel grid was defined as a selection point for the feature extraction of the brain MRI images. Variations in pixel size affect the computational cost and feature vector size. Furthermore, the four-element vectors ([16, 32, 48, 64] and [17, 34, 51, 68]) were used to extract the KAZE and SURF features, respectively. The details of KAZE and SURF extraction were already provided in Section 2.1. After this, 20% of the redundant features were discarded to reduce the feature vector size. Finally, based on the simplicity and robustness, the *k*-means clustering algorithm was utilized for feature segmentation. Furthermore, it kept observations inside each cluster as close to each other and as far away from objects in other clusters as possible Therefore, 400-feature histograms were created using the *k*-means clustering approach. Further details about the *k*-means clustering approach can be found in [34,35]. After this, various machine learning classifiers, such as SVM, tree [36], Naïve Bayes [37], k-nearest neighbors (K-NN) [38], ensemble, and neural network (NN), were used to train the models. The results of the proposed method are presented in the subsequent section.

## 3. Brain MRI Dataset and Results

**Figure 2:**
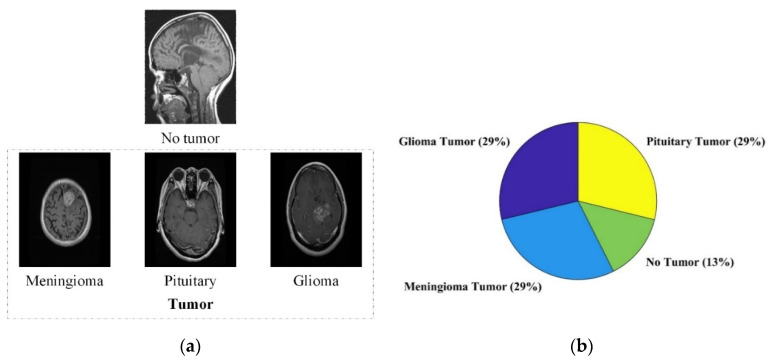
(**a**) The brain MRI images of each class; (**b**) the percentage distribution of MRI images per class.

This study validates the suggested paradigm using an online collection of brain MRI images [39]. The dataset for this study was obtained from the Kaggle website [39]. It contains three tumor classes (glioma, pituitary, and meningioma) and one class of no tumor. It has 2870 brain MRI images in total. Additionally, 80% of the data of each class were utilized for the training of the models. The remaining 20% of the data were used to test the trained models. The brain MRI images and percentage distribution of images per class are shown in Figure 2.

**Figure 3:**
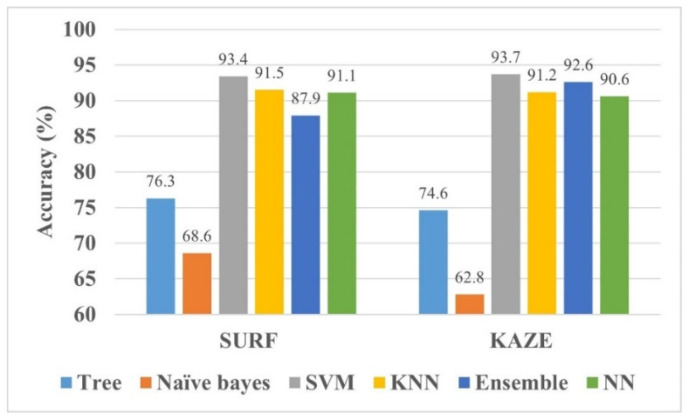
The comparison of various machine learning models for SURF and KAZE features.

In this work, MATLAB 2021 was utilized for training the models in the 64-bit Windows 11 operating system (core i7, 11th generation, 32 GB RAM, NVIDIA GeForce GTX 1060, and 1 TB SSD). In addition, the classification accuracy was used as a comparison metric for the various trained models (SVM, tree, Naïve Bayes, K-NN, ensemble, and NN). The results of the KAZE- and SURF-trained models are presented in Figure 3.

**Figure 4:**
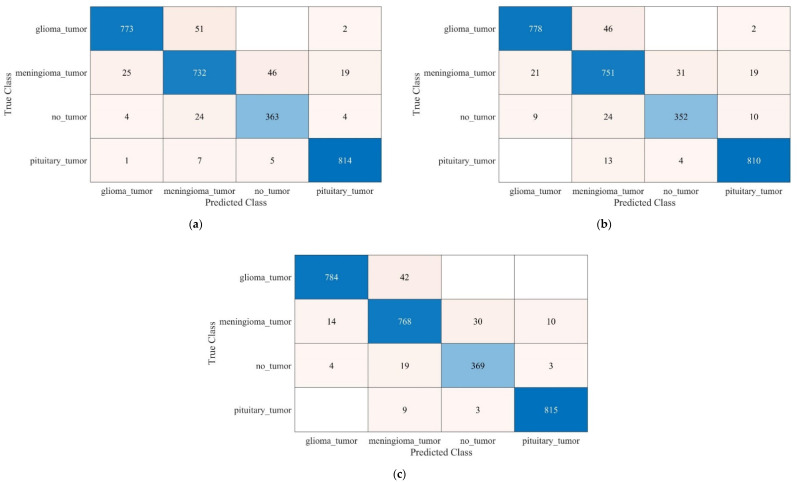
Confusion matrixes of various models: (**a**) SURF-trained SVM; (**b**) KAZE-trained SVM; (**c**) SURF + KAZE (hybrid)-trained SVM (proposed model).

It is evident from Figure 3 that the SVM model trained with SURF and KAZE features shows accuracies of 93.4% and 93.7%, respectively, which are the highest among all methods. Therefore, it may be fruitful to concatenate the features of SURF and KAZE to determine the model’s performance in classifying brain MRI images. Furthermore, the confusion matrixes of the SURF-, KAZE-, and SURF + KAZE- (hybrid) trained SVM models are shown in Figure 4.

**Figure 5:**
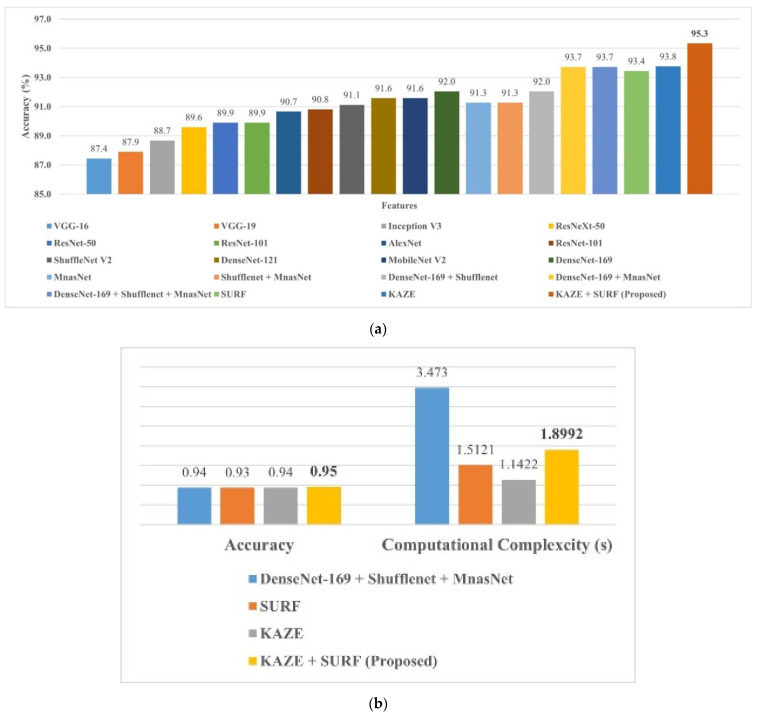
Comparison of SVM model trained with deep features with the proposed model: (**a**) accuracy comparison; (**b**) accuracy and computational complexity.

The SVM model trained with concatenation features shows the highest accuracy of 95.33%, almost 2% higher than the SVM model trained with SURF features. Therefore, the proposed SURF + KAZE-trained SVM model has true positive rates (TPRs) of 97.75% and 98.42% for the glioma and pituitary tumor classes. Furthermore, the proposed model correctly classifies 19 more MRI brain images for the no tumor class than the KAZE-trained model. Similarly, 36 more brain MRI images are correctly classified for the meningioma tumor class compared to the SURF-trained model. Finally, the proposed model is compared with the pre-trained deep-feature-trained SVM model presented by Kang et al. [25]. The comparison results of various SVM models are presented in Figure 5.

**Figure 6:**
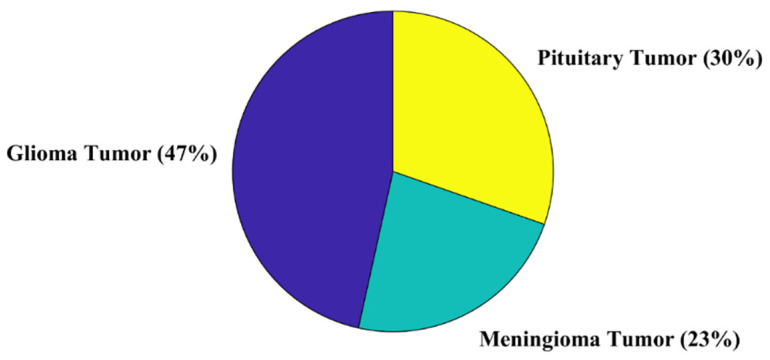
The percentage distribution per class of brain MRI dataset [40].

**Figure 7:**
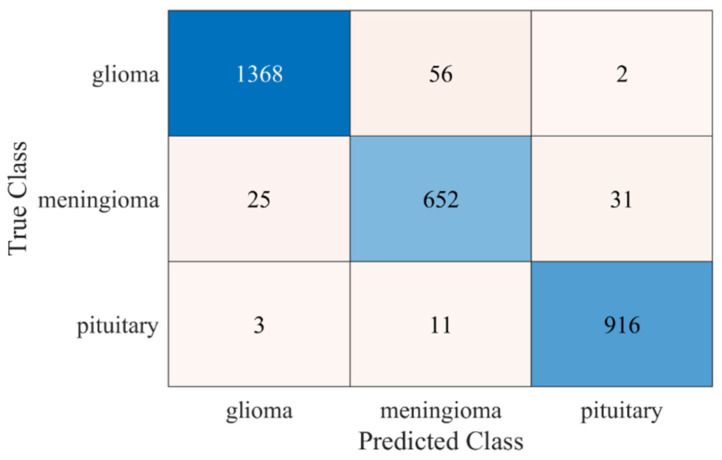
Confusion matrix of the proposed model for new dataset [40].

For further validation of the proposed approach, another public dataset is utilized [40]. The dataset contains a total of 3064 brain MRI scans. Further details about the dataset are shown in Figure 6. The classification result of the new dataset is presented in Figure 7.

## 4. Discussion

Computer-aided detection/diagnosis involves a computer-based system that assists clinicians in making quick judgments in the field of medical imaging. Several studies have reported several training methods for categorizing brain MRI images [8,16,22,25,41].

In this work, an SVM model for brain MRI images trained with hybrid SURF and KAZE features is proposed for brain tumor classification. First, the acquired brain MRI images were processed using the 8 × 8-pixel uniform grid to extract the SURF and KAZE features, as discussed in Section 2.1.1 and Section 2.1.2. As a result, 16,577,120 features were extracted for the whole dataset containing 2870 brain MRI images of various classes (see Section 3 for details). In addition, 80% of the strongest features were computed using the computer vision toolbox of MATLAB, which reduced the feature vector size to 7,300,864 for all of the brain MRI images. Finally, *k*-means clustering was utilized to form feature vectors with a size of 400 for each image. As a result, the SVM-trained model showed the best accuracies of 93.4% and 93.7% for SURF and KAZE, respectively (see Figure 3). Furthermore, the concatenation of both the SURF and KAZE features resulted in a better accuracy of 95.3% for brain MRI multiclass classification.

Kang et al. [25] trained the SVM model using pre-trained network deep features. The results suggested that the DenseNet-169 + Shufflenet + MnasNet-trained SVM model had the best classification accuracy of 93.72% for a similar dataset (see Figure 5). The proposed SURF + KAZE-trained SVM model showed an accuracy of 95.33%, almost 1.5% higher than the model proposed by Kang et al. (see Figure 5a). The computational cost of the proposed model was also almost two times lower than their proposed model (see Figure 5b). In a study [41], pre-trained CNN models (GoogleNet, VGGNet, and AlexNet) were utilized to classify brain MRI images. The model showed high classification accuracy with a high training time of around 1 h and 30 min for the fine-tuned VGGNet CNN model. The model presented in our study (SURF + KAZE) showed an accuracy of 95.33% and had a computational complexity of only 1.8992 s. For further validation, a new public dataset that had three classes was used to check the performance of the proposed framework (see Figure 6). The proposed approach showed similar accuracy (95.9%) for the classification in the new brain MRI dataset, as shown in Figure 7. The results validate the adeptness, robustness, and high classification accuracy of the proposed approach. This demonstrates that the presented model is relatively straightforward to implement for real-time applications. As a result, the suggested technique has the potential to play a critical role in assisting clinicians/doctors for early brain cancer detection.

## 5. Conclusions

This study presents an automatic brain tumor diagnostic approach using brain MRI images. First, the proposed approach computes the SURF and KAZE features using a grid of 8 × 8 pixels in size of brain MRI images. Then, 80% of the strongest features are considered for segmentation using *k*-means clustering. The final feature vector has a size of 400 per image for each feature (SURF and KAZE). Finally, the proposed hybrid feature vector is used to train the SVM model. The classification accuracies of the proposed model (SURF + KAZE) are 95.33% and 95.9%, almost 2% higher than the SURF-trained SVM model. The comparison of the proposed approach with the findings presented in the literature also shows its superiority due to its high accuracy and lower computational time. Thus, the proposed approach can be used for the automatic detection of brain tumors.

## Data Availability

Not applicable.
